# Child and Family Outcomes and Experiences Related to Family-Centered Care Interventions for Hospitalized Pediatric Patients: A Systematic Review

**DOI:** 10.3390/children11080949

**Published:** 2024-08-06

**Authors:** Christine R. Hodgson, Renee Mehra, Linda S. Franck

**Affiliations:** School of Nursing, Family Health Care Nursing, University of California, San Francisco, CA 94143, USA; christine.hodgson@ucsf.edu (C.R.H.); renee.mehra@ucsf.edu (R.M.)

**Keywords:** systematic review, family-centered care, pediatric hospitalization, child, parent, satisfaction, hospital experience, parent–staff relationships

## Abstract

Background/Objectives: Family-centered care (FCC) is the recommended model for pediatric inpatient care. Our overall aim was to conduct a narrative synthesis of the contemporary published research on the effectiveness of FCC interventions for pediatric inpatients. Our specific objective was to critique studies of inpatient pediatric FCC interventions that evaluated child or parent outcomes. Methods: We searched five databases (Pubmed, CINAHL, Embase, PsychInfo, and Web of Science) for peer-reviewed research published from 1 January 2017 to 6 February 2024. Independent reviewers evaluated each study based on pre-specified inclusion and exclusion criteria, then extracted and narratively synthesized the data. Results: We found 16 studies of 15 interventions conducted in six countries. The studies were quantitative (*n* = 11), qualitative (*n* = 3), and mixed methods (*n* = 2), with most designs being of low to moderate quality based on a modified Mixed-Methods Appraisal Tool. Interventions included family-centered rounds, parent-focused health information technology, education, patient navigation, parent–peer support, partnership, and parent participation in caregiving. Most studies found significant improvements in parents’ well-being, knowledge, and participation, as well as decreased stress and anxiety with the FCC interventions compared to usual care. One study found no differences in child outcomes (infant feeding, length of stay) between usual care and a parent-participation intervention. Conclusions: Although FCC interventions led to many improved outcomes for parents, there were few well-designed comparison studies using validated tools and well-defined interventions. Higher quality research is needed to promote greater uptake and sustainability of FCC interventions globally.

## 1. Introduction

Family-centered care (FCC) is a strengths-based approach to health care that involves the full partnership of the patient and their family in all aspects of health care delivery at the individual, institutional, and health system levels [1,2,3]. In some countries, the term “humanization” is used to describe FCC [4,5]. FCC in pediatrics is delivered according to the four core principles of respect and dignity, information sharing, participation, and collaboration [6,7] (see Table 1). We use the term “parent” to include mothers, fathers, or other primary caregivers. FCC is based on a foundation of mutual trust and power sharing between families and the healthcare team [8]. For over a decade, FCC has been the recommended model for pediatric inpatient care in many countries, with the World Health Organization including FCC in their standards for the quality of healthcare for children [9]. However, consensus is lacking regarding the specific interventions that lead to the desired outcomes and care delivery experiences within the FCC model.

Three reviews summarize the global research over the past two decades on FCC interventions in pediatric hospital settings [4,10,11]. Segers et al. [11] systematically reviewed the FCC intervention research published between 2003 and 2017. They found 17 studies published in Dutch, English, French, and German (the countries were not reported) in the Pediatric Intensive Care Unit (PICU) (*n* = 4) and Neonatal Intensive Care Unit (NICU) settings (*n* = 13), with 4742 pediatric or neonatal patients. The study designs included randomized controlled trials (RCTs) (*n* = 3), quasi-experimental (*n* = 12), cross-sectional (*n* = 1), and observational (*n* = 1). The most common types of FCC interventions were parent–participation and parent–staff collaboration, and the most common outcomes were length of stay (LOS) and parent satisfaction. Four studies of moderate to high quality (three in NICUs and one in a PICU) found that family-centered rounds (FCRs) or parent participation in direct caregiving interventions led to a significantly shorter length of stay (LOS) of between 3.8 to 19 days (*p* < 0.05) compared with usual care [12,13,14,15]. Eight studies of low to moderate quality reported significantly higher parent satisfaction after interventions focused on parent participation, FCRs, communication, or rooming-in (all *p* < 0.05 or less) compared with usual care [12,16,17,18,19,20,21,22]. No adverse effects were reported in any of the studies. Segers et al. [11] concluded that there was moderate to strong evidence for FCC interventions to improve parent satisfaction and infant LOS in the NICU setting. However, the PICU studies were too few and of insufficient quality to determine the relationships between parent participation or parent–staff collaboration interventions and LOS or parent satisfaction outcomes. 

Tripodi et al. [4] systematically reviewed research published on humanization interventions between 2000 and February 2018. The authors included 28 studies with 3345 parents of hospitalized children conducted in general pediatric units in the United States (US, *n* = 15); Italy (*n* = 4); Canada, Iran, and Israel (*n* = 2 in each country); Iceland, Mexico, and South Africa (*n*= 1 in each country). The study designs were quasi-experimental (*n* = 21) or cross-sectional (*n* = 7), and most were single-center studies. Six of the 28 studies (21%) were rated high quality. The studies evaluated a range of humanization interventions related to the concepts of respect and dignity (providing psychological support and pet therapy), information sharing (FCRs), parent partnership (technology and environmental change to promote parental engagement), and collaboration (parent–staff collaboration). Nineteen studies reported significant improvements in a variety of child and parent outcomes and no safety concerns compared to controls (*p* < 0.05 or less). Examples of significant findings were FCR interventions associated with increased timely hospital discharge and parent engagement with FCR interventions, decreased child pain, anxiety, or need for sedation with pet therapy or hospital clowns, and increased social need screening or increased parent satisfaction with parent-focused technology interventions. Seven studies reported high parent satisfaction with the interventions, although there were no significant differences compared with usual care. Tripodi et al. [4] concluded that the interventions were mostly effective, the humanization models were not well-specified, and humanization in pediatric units requires further research. 

Phiri et al. [10] performed an integrative review of research published between 2008 and 2018 on FCC interventions in developing countries [23]. Eleven studies, with a total of 1306 parents of hospitalized children, were included from Iran (*n* = 4), Jordan (*n* = 2), South Africa (*n* = 2), India (*n* = 1), Nigeria (*n* = 1), and Pakistan (*n* = 1), in the NICU (*n* = 3), PICU (*n* = 3), pediatric wards (*n* = 4), or emergency rooms (*n* = 1). The study designs were RCT (*n* = 4), quasi-experimental (*n* = 1), cross-sectional (*n* = 5), or qualitative (*n* = 1). All studies were assessed as moderate quality. The studies reported that FCC information sharing and parent participation interventions were associated with improved health outcomes, including decreased LOS (*n* = 3), decreased readmission (*n* = 3), improved neonatal feeding at discharge (*n* = 1), and improved maternal attachment and parent satisfaction (*n* = 1) (all *p* < 0.05 or less) compared with usual care. While most results across studies showed significant improvements or no change in outcomes, there was one study where the parents had mixed satisfaction scores across domains of an FCC instrument [23]. Phiri et al. [10] concluded that the studies had inconsistent descriptions of the FCC elements in each intervention, and the methodology and outcome measurements varied greatly, limiting the overall conclusions about the strength of the evidence for the interventions. 

In summary, the prior reviews of FCC interventions show that most of the studies have focused on the neonatal population, are of moderate quality, and demonstrate that a range of FCC interventions have a positive effect on parent outcomes (e.g., satisfaction with care and infant outcomes (e.g., hospital LOS)) compared with usual care [11]. Fewer studies have focused on FCC for older hospitalized children. These studies are of low to moderate quality, with varied methods and outcome measures. Thus, more research is needed about the effectiveness of FCC interventions in inpatient pediatric settings [4,10]. Given the publication dates for the studies included in the three prior related systematic reviews, we focused our analysis on research published since those reviews. 

### Objective

Our overall aim was to conduct a narrative synthesis of the contemporary published research on the effectiveness of FCC interventions for pediatric inpatients. Our specific objective was to critique studies of inpatient pediatric FCC interventions that evaluated child or parent outcomes. 

## 2. Materials and Methods

### 2.1. Study Design 

We conducted a systematic review following the PRISMA guidelines [24]. See Supplementary Materials S1 for PRISMA checklists.

### 2.2. Search Strategy

We included quantitative, qualitative, and mixed-methods studies published in English between January 2017 and February 2024 from Pubmed, CINAHL, Embase, PsychInfo, and Web of Science. We conducted our initial search on 14 February 2023 and updated the search on 6 February 2024. See Supplementary Materials S2 for search strategies for each database. See Table 2 for the details of the inclusion and exclusion criteria. We imported 1442 title/abstract results from the five databases into Covidence Software [25], which identified 528 duplicates that we removed from the dataset.

### 2.3. Selection of Studies

**Figure 1:**
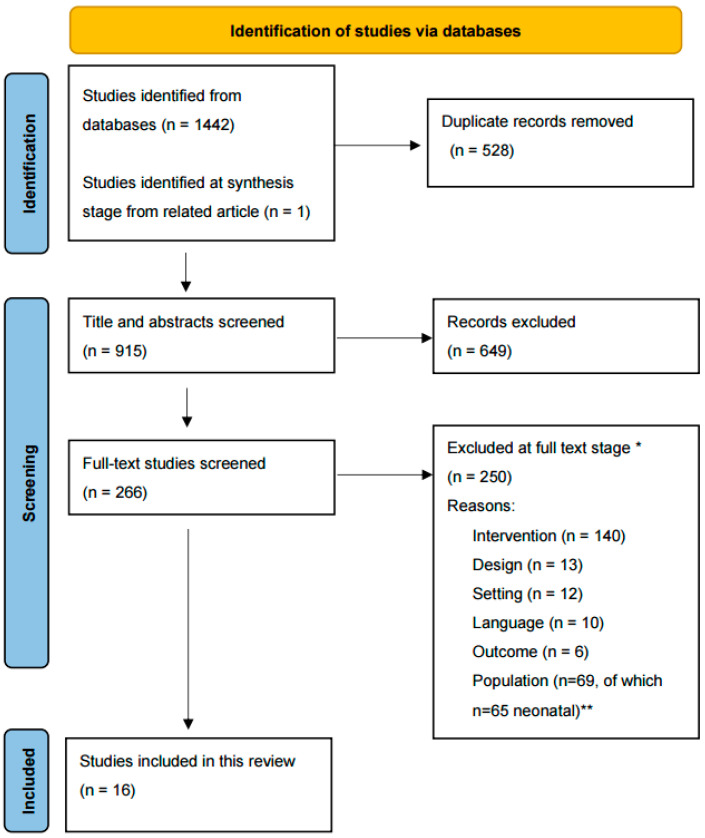
Preferred Reporting Items for Systematic Reviews and Meta-Analysis Diagram [24]. * Full-text studies were allowed multiple reasons for exclusion based on a hierarchy of exclusion codes. Only the highest-ranked exclusion reasons for each study are reported here. ** Of studies excluded for population, those with a neonatal population (*n* = 65) were included in the initial search strategy but later excluded from this systematic review for separate analysis and reporting.

Two of three reviewers (CH, RM, LF) independently assessed 915 titles and abstracts to identify and exclude irrelevant studies. Where there was disagreement between reviewers, the selection was determined by consensus of the three reviewers. The full texts of 266 articles were then retrieved for the screened studies. The reviewing strategy was repeated with two of three reviewers (CH, RM, LF) independently, examining the full text of the studies and determining inclusion by consensus of the three reviewers when there were disagreements. Studies were excluded if they were irrelevant, based on an assessment of the full text. Most excluded studies were eliminated because they did not report on a well-defined FCC intervention. The studies were unevenly distributed across pediatric inpatient settings. Two-thirds of the studies (*n* = 65) were conducted in the neonatal intensive care (NICU) unit with neonates and their families, and the remaining studies (*n* = 16) were conducted in other inpatient pediatric settings with infants, children, and their families. As a result of the number of FCC intervention studies in the NICU setting, we determined that this body of research warranted a separate systematic review. Therefore, we excluded the NICU studies from this review and included only the 16 pediatric-focused FCC studies. See Figure 1 for details.

### 2.4. Data Collection Process

We developed an extraction tool to organize and categorize data from each study. Two authors (CH and RM) independently extracted data using a standard spreadsheet and compared the results. We discussed differences until we reached a consensus. Some studies had multiple objectives irrelevant to our research aim, which were not extracted or synthesized. For example, we did not evaluate outcomes related to healthcare staff or health systems. Similarly, descriptive studies about FCC that did not involve an intervention were excluded, since our focus was on evaluating the effectiveness of FCC interventions. We identified the most highly emphasized FCC principle for each intervention to organize the reporting of interventions in this review.

### 2.5. Quality Appraisal

We conducted a quality appraisal using our own adaptation of the mixed-methods appraisal tool (MMAT) [26]. The MMAT was developed to critically appraise five study methodologies: RCTs, non-randomized studies, quantitative descriptive studies, qualitative research, and mixed-methods studies. We assigned each study a score based on the MMAT updated scoring guidance [27] and on a customized scoring system we developed to calculate a percentage of criteria met (see Supplementary Materials S3). Two independent authors (CH and RM) appraised each study, and differences were discussed until we reached a consensus.

### 2.6. Synthesis

The quantitative and qualitative findings are jointly reported in a narrative synthesis to map the collective knowledge in relation to the FCC principles [28]. We organized the FCC intervention in each study by the core principles of respect and dignity, information sharing, participation, and collaboration. We refer to both qualitative studies and qualitative strands of mixed-methods studies as “qualitative” for simplicity. We note effect sizes in the narrative synthesis if reported by the authors and all significant findings of *p* < 0.05 or less. For the final synthesis, we evaluate and discuss the qualitative themes in relation to the quantitative results.

## 3. Results

### 3.1. Study Characteristics 

We found 16 studies that evaluated 15 interventions designed to promote one or more dimensions of FCC in pediatric inpatient settings. The studies took place in the US (*n* = 9) [29,30,31,32,33,34,35,36,37], the US and Canada (*n* = 1) [38], Canada (*n* = 1) [39], Korea (two studies on one intervention) [40,41], Indonesia [42], Iran [43], and Switzerland [44] (*n* = 1 in each country). The settings included PICUs (*n* = 3) [29,34,39], a pediatric cardiac ICU (PCICU; *n* = 2 studies on one intervention) [40,41], pediatric surgical, medical, subspecialty or general units (*n* = 8) [30,31,32,33,36,38,43,44], the pediatric emergency department (*n* = 1) [35], pediatric hematopoietic stem cell transplant unit (*n* = 1) [37], and a pediatric oncology unit (*n* = 1) [42]. The 16 studies included a total of 3801 parents of hospitalized children (range: 10 to 1864 participants. Nine studies reported on parents’ gender, ranging from 76% to 83% of the total sample identifying as female [31,32,33,34,35,36,37,38,44]. No studies reported gender identities other than male or female. Four studies purposely limited the sample to mothers [40,41,42,43], and three studies did not report on either gender or the participants’ relation to children [29,30,39]. The only study that measured outcomes for children reported that 50% of the children in the intervention group were female, and 46% of the children in the control group were female [41].

The quantitative study designs were quasi-experimental (*n* = 8) [29,33,34,38,39,41,42,43], observational cross-sectional (*n* = 2) [31,32], and observational longitudinal (*n* = 1) [40]. There were three qualitative studies, two using interview methods [30,37] and one using a retrospective design with a narrative analysis of family partner notes from medical record [36]. Two mixed-methods studies included one cross-sectional study using surveys, focus groups, and interviews [35] and one quasi-experimental using surveys plus interviews [44]. See Supplementary Materials S4 for a summary of the study characteristics and main FCC findings and Table 3 for details about the outcome variable measures used in the studies.

### 3.2. Quality Appraisal of Studies

The average quality score for the studies was 64%, ranging from 36% to 100% (see Supplementary Materials S2). Common strengths in the quasi-experimental and cross-sectional studies were (a) having clear inclusion and exclusion criteria, (b) having measurements that were clear, justified, and appropriate for answering the research question, and (c) having measurements that reflect what they are supposed to measure. The weaknesses in quasi-experimental and cross-sectional studies were an absence of reporting on the following: (a) reasons why certain eligible individuals chose not to participate, (b) attempts to achieve a sample of participants that represents the target population, (c) non-response rate and reason for non-response, (d) having complete outcome data, (e) justification of statistical analyses used, and (f) accounting for confounders in the design and analysis. Although the MMAT scoring did not emphasize the type of statistical analysis, only three studies used advanced analyses that could control for potential confounders: regression analysis [33,38] and ANCOVA [41].

The strengths of the five qualitative studies were consistent interpretation of results supported by the data collected and sufficient quotations provided to justify the themes. The weaknesses among the qualitative studies included inadequate data (such as not reporting on the use of field notes and memos) and lacking clear links between data sources, collection, analysis, and interpretation. We found the two mixed-methods studies to be strong in reporting when the integration of quantitative and qualitative data occurred but weak in describing how the discordant findings were addressed. Mixed-methods studies may have received lower quality scores because, with the MMAT, the overall quality of a study cannot exceed the value of the study’s lowest-scoring component [27].

### 3.3. Study Findings by FCC Principle and Intervention Type

We present a summary of key findings from the 16 studies below, organized by the FCC principle that most closely matched the intervention. The most common principles were information sharing interventions (*n* = 8), followed by respect and dignity interventions (*n* = 4), and parent participation interventions (*n* = 2). Two studies about one intervention gave equal emphasis to more than one principle, so we considered those studies as having a mixed principle focus [40,41]. The principle of collaboration was demonstrated in the partnership with parents (or patient and family advisory committees) to develop interventions or outcome measures in nine studies [29,30,33,36,37,38,39,40,41]. In these studies, the researchers used various methods to engage parents in developing the intervention, including surveys, interviews, focus groups, pilot tests, and embedding parents into the program as volunteers or paid staff. However, the measurements or outcomes of the collaboration were not explicitly reported; thus, no results on FCC collaboration interventions are reported in this review.

#### 3.3.1. Information Sharing Interventions

Eight studies evaluated information sharing interventions to promote FCC, including FCRs (*n* = 3) [31,33,38], education for parents (*n* = 3) [29,42,43], and technology interventions to improve parents’ access to information (*n* = 2) [32,37].

##### FCR Interventions

In the most extensive study of an FCC intervention in this review, Khan et al. [38] conducted a quasi-experimental study of an FCR program on seven pediatric floors of U.S. hospitals (*n* = 947 parents in the pre-intervention group; *n* = 890 in the post-intervention group) and reported a significant decrease in harmful medical errors by 37.9% from pre- to post-intervention. The non-harmful errors did not differ pre–post intervention. Significant improvement was also noted in five measurements of parents’ roles during FCRs (top-box “excellent” scores increased by 11.1 to 21.8 percentage points) and six of 25 domains of parent experience on a self-developed family experience scale (top-box “excellent” ratings increased by 7.5 to 18.1 percentage points). There were no significant changes in many parent-experience item scores, but no scores worsened. Unadjusted results were reported as the distributions of potential confounders were comparable between pre- and post-intervention groups.

In another quasi-experimental study of an FCR intervention [33], a nighttime communication intervention for families and staff was evaluated in two U.S. pediatric units (*n* = 281 parents in the pre-intervention group; *n* = 183 parents in the post-intervention group). The intervention included a nurse–physician briefing, a family huddle, and a family update sheet. The shared understanding between parents and nurse ssignificantly increased from pre- to post-intervention by 12 percentage points (top-box scores), and parents’ experience communicating with nighttime doctors increased by 7.9 percentage points from pre- to post-intervention. No outcomes worsened, and there was no change in shared understanding between parents and residents nor in the number of top-box responses for three other parent-experience items, adjusted for confounders. The third FCR study was a cross-sectional study on a US children’s hospital pediatric unit where parents were given FCR written information via paper, tablet, or computer [31]. Only descriptive results were reported. Parents (*n* = 200) completed surveys about factors contributing to their engagement during FCRs: having clear explanations from the medical team (78.5%), understanding medical information (75.5%), factors dependent on the health of the child (74.5%), and the medical team asking their input (71%). Half of the parents reported no preference between using paper, tablet, or computer resources for FCRs. Among the parents who reported a preference, those exposed to tablets had a 27-percentage point increase in having a resource preference for tablets compared to those who were not exposed.

##### Educational Interventions

Nankali et al. [43] implemented an FCC educational program to increase mothers’ knowledge about participating in their child’s care for acute gastroenteritis while on a general pediatric unit in Iran. The intervention consisted of one to three individualized education sessions with mothers. The quasi-experimental study compared caregiving scale scores (*n* = 80 mothers, pre-intervention control; *n* = 80 different mothers, post-intervention) and found that the mean caregiving scores improved more than 3-fold for the intervention group from pre-test to post-test. Post-test mean difference scores were higher for the intervention group compared to the control group. In contrast, the control group scores did not improve between pre- and post-test, and the pre-test mean scores did not differ between the intervention and control groups. Unadjusted results that were reported as potential confounders were comparable between the intervention and control groups. Chamblee and Miles [29] conducted a study among parents of children with central lines in a US hospital, who received written information about how parents could partner with staff to prevent central line-associated bloodstream infections (CLABSI). In the quasi-experimental study (*n* = 59 parents, pre-intervention; *n* = 62 different parents, post-intervention), the post-intervention group had significant improvements in all four survey items measuring CLABSI knowledge and prevention compared to the pre-intervention group. Parents in the post-intervention group rated FCC as occurring more consistently than parents in the pre-intervention group across all four FCC principles. However, confounders were not accounted for in the design and analysis, which may either under- or over-estimate the relationship. In the final FCC educational intervention, Krisnana et al. [42] used a quasi-experimental design to evaluate an educational FCC module that included in-person teaching sessions for mothers of children hospitalized in an Indonesian pediatric oncology unit to learn about their child’s care (*n* = 30 mothers in the intervention group; *n* = 30 mothers in the control group). The post-test stress scores were 35% lower in the intervention group compared to the control group. However, confounders were not accounted for in the analysis, so these findings should be interpreted cautiously.

##### Parent-Focused Health Information Technology

Kelly et al. [32] conducted a cross-sectional study in a US pediatric unit to evaluate a patient portal intervention that enabled parents to access real-time information about their child’s hospital stay from an electronic health record (EHR). A post-intervention survey of 90 parents who used the portal found that 90% of the parents were satisfied with the portal, 89% reported they felt the portal would reduce errors in care (8% of the parents found actual errors in their child’s medication list), and 60% thought that the portal improved healthcare communication. In addition to descriptive statistics, comparisons were made regarding satisfaction among parents with different characteristics. Parents with lower levels of education or who had not previously used an outpatient portal were more satisfied with the portal.

In another study [37], the parents of children undergoing bone marrow transplant (BMT) in a US pediatric unit were provided access to an electronic “BMT Roadmap” with links to EHR lab results for their child, a healthcare team directory, phases of care information and a discharge checklist. In qualitative interviews (*n* = 10) after the implementation of the BMT Roadmap, the parents reported that the tool was useful and easy to use and led them to want even greater access to information. They discussed the emotional impact of the BMT process, the critical importance of communication among patients, caregivers, and healthcare providers, ways in which the BMT Roadmap was helpful, and other strategies for the organization and management of complex healthcare needs that could be incorporated into the BMT Roadmap.

#### 3.3.2. Respect and Dignity Interventions

Four studies investigated interventions that promoted respect and dignity for parents. These included two studies of peer support [36,39] and two studies of healthcare navigation interventions led by professionals [30,44].

##### Parent–Peer Support Interventions

The FCC peer-support interventions were evaluated in two studies. We noted that these two studies had the lowest quality scores of all 16 studies (36% [39] and 38% [36]). Pollock et al. [36] investigated a peer-support program for parents of children and youth with special healthcare needs (CYSHCN) in ambulatory and inpatient settings of a US children’s hospital using a mixed-methods study design. The intervention included social, emotional, cultural, and educational support from peers who had lived experience caring for a CYSHCN and were trained and hired as family partners. Qualitative data were collected from a retrospective chart review of the family partner notes. For the participating parents (*n* = 28), there was EHR documentation of 100 supportive contacts with family partners, with 78% of the contacts providing emotional support and 38% providing tangible support. The common themes identified were the complexity of caring for CYSHCN, the need for stress management, financial concerns, health concerns, and the need for parent self-care. The lack of reporting about adequate sampling methods, measures, and analysis in the quantitative component of the study resulted in a lower quality score. In a second study, Pereira et al. [39] developed a two-pronged FCC support intervention for parents whose children were in a Canadian PICU by assigning them a peer volunteer who was over 18 years old and had a personal history of hospitalization (*n* = 25 parents) or a peer mentor who had a similar lived experience of having a child in the PICU (*n* = 21 parents). Pre-intervention, 84% of the parents were positive about the idea of the peer volunteers and 52% about the peer mentors. The COVID-19 pandemic response disrupted the study. Few participants completed the post-intervention surveys (volunteers, *n* = 5 and peer mentors, *n* = 6), and most surveys were incomplete. Post-intervention, the parents’ rating of benefits was neutral to positive. One parent (25%) rated the volunteers negatively. Only descriptive statistics were presented. Lack of reporting about adequate sampling methods, measures, and analysis brought down the quality score of this quantitative study.

##### Healthcare Navigation Programs Led by Professionals

The qualitative interview study by Chisholm et al. [30] of an FCC patient navigation program for parents of low-income children of color who were hospitalized for multiple conditions in a US pediatric unit achieved a perfect score in our quality appraisal (*n* = 50). A bilingual (Spanish) professional family guide led the program and provided education about the hospital, a social needs assessment, a cultural and communication assessment, communication coaching for parents, emotional support through frequent check-ins, and a follow-up phone call about their experience. The parents described themes of improved communication, feeling supported in the care environment, and increased knowledge, skills and understanding of the hospital and resources after participating in the intervention.

In another study, a parent navigation program provided preadmission support for parents of children with profound disabilities undergoing surgery in two pediatric surgical units in Switzerland [44]. An advanced practice registered nurse (APRN) provided written information, a counseling phone call before surgery, at least one hospital visit, and support for the healthcare team. The intervention was evaluated with a quasi-experimental, mixed-methods study (*n* = 14 parents in the intervention group; *n* = 14 parents in a historical control group). There were no significant differences in parent satisfaction between the intervention and control groups. There were non-significant lower scores in four domains and higher scores in one domain of parent satisfaction. The results were not adjusted for confounders despite the demographic differences between the two groups, so these findings should be interpreted with caution. The qualitative findings indicated that parents felt well-prepared and appreciated the preadmission intervention. However, some had unmet expectations that the entire hospital course would be similarly organized and reported that the remainder of their hospital experience fell short of their expectations, with some mentioning feeling disregarded or unappreciated by hospital staff. The study received a low-quality score due to an inadequate rationale for using a mixed-methods design, lack of integration of the qualitative and quantitative components of the study or discussion of the divergences between the quantitative and qualitative components.

#### 3.3.3. Interventions to Promote Parent Participation in Direct Caregiving

Two studies evaluated different parent participation interventions [34,35].

##### Physical Touch and Holding

Leland et al. [34] evaluated an intervention enabling parents to hold their children in a US PICU. All children, including those mechanically ventilated, were eligible for holding by their parents. The PICU provided multidisciplinary training to the staff, as well as written information and personal support to parents. The quasi-experimental study compared caregiver spiritual well-being scores (*n* = 174 parents, pre-intervention; *n* = 157 different parents, post-intervention) before and after the implementation of the intervention. The day-four caregiver spiritual well-being scores were assessed on the fourth day after enrollment and were higher for the post-intervention group compared to the pre-intervention group (an effect size of 0.47). There was at least a 20-percentage point increase for parents in the intervention group who reported physical contact with their child on perceptions of team support for physical contact with their child, sense of being valued as a member of the team, and the child’s illness being less of a barrier to physical contact. The caregiver spiritual well-being scores on the day of enrollment and on safety (measured by unplanned extubation rates) were not different between the groups. Unadjusted results were reported; potential confounders were comparable between pre- and post-intervention groups or were not associated with caregiver spiritual well-being.

##### Parents’ Presence during Their Child’s Resuscitation

O’Connell et al. [35] performed a quasi-experimental mixed-methods study of an intervention enabling parents to be present during their child’s resuscitation in three level-1 trauma pediatric emergency departments in the US. All staff received training, and one social worker was assigned to each parent, regardless of presence. The researchers administered surveys, focus groups, and telephone interviews both with the parents who were present during their child’s resuscitation (*n* = 99) and with the parents who were not present for any reason (*n* = 27), at 3 to 6 months after the child was discharged. Only descriptive statistics were presented. Almost all (90–94%) parents who were present reported positive interactions that included providing emotional support, talking to, being near, and touching their child. Most (81–92%) recalled having an interactive relationship with the trauma team. Almost all (90–100%) parents who were present agreed with statements about the importance of being with their child, wanting to be in the room with their child again during medical care, how being there lowered child and parent anxiety, having a better understanding of their child’s condition, and having the right to be present. Of the 27 parents not present, the reasons were the following: not physically present (*n* = 15), no reason (*n* = 7), not allowed for team-related reasons (n = 4), and choosing not to be present (*n* = 1). The parents who were not present were interviewed with hypothetical wording (e.g., “would have been important”), and they agreed with the statements above to a lesser degree (52–82%). The qualitative findings corroborated the descriptive survey responses for both groups of parents. Common themes included parental views about their right to be present, limitations to being there, benefits to the child, including advocacy and comfort, peace of mind and comfort for the parent, the importance of seeing the child’s care in real-time, and how presence promoted trust with the staff. This study also received a low-quality score due to an inadequate rationale for using a mixed-methods design, lack of integration of the qualitative and quantitative components of the study, or discussion of the divergences between quantitative and qualitative components.

#### 3.3.4. Multiple-Principle FCC Interventions

Two studies about one intervention evaluated information sharing and parent participation [40,41].

##### Information Sharing and Parent Participation

Two studies evaluated a mother–nurse partnership intervention that aimed to increase both information sharing and parent participation. The mothers of hospitalized children in a cardiac PICU in Korea received 30 min education sessions twice daily and written information. They were encouraged to participate in child caregiving activities such as feeding, hygiene, and changing diapers. The first quasi-experimental study compared mothers who received the intervention (*n* = 36) to mothers in a control group who received regular nursing care (*n* = 37) [41]. In adjusted analyses, from pre- to post-intervention, maternal anxiety decreased by 30% in the intervention group and 12% in the control group. In the intervention group, parental self-efficacy increased by 21% (compared to 6% in the control group), parental satisfaction increased by 17% (compared to 2% in the control group), and perceived partnership increased by 14% (compared to 1% in the control group). There were no differences in infant outcomes of time to reach full oral feeding and length of stay. Uhm and Choi [40] performed a cross-sectional secondary analysis of data from the same mother–nurse partnership intervention and measured outcomes of mothers in the intervention group (*n* = 36) as their infants progressed through five recovery phases (postoperative, early ventilator weaning, late ventilator weaning, post-extubating, and transfer preparation). The mothers’ duration of participation significantly increased at each phase of recovery, with participation time doubling from the first to the fifth phase. The ratings of the mothers’ physical engagement and psychological connectedness with their child also significantly increased, approximately doubling, over the infant recovery phases.

### 3.4. Synthesis of Findings

#### 3.4.1. Quantitative Synthesis

The most common study design was quasi-experimental, usually with different cohorts, pre- and post-intervention groups. The cross-sectional studies conducted descriptive rather than analytical analyses, so there were no comparative analyses. Constraints of time, space, cost, and ethical considerations were typically noted as reasons for the choice of study design. Different outcomes were measured in each study, precluding direct comparison of findings across studies (see Table 3). The authors developed new instruments in eight studies to measure the outcomes [29,31,32,33,36,38,39,40]. In two studies, the authors developed new measures and used previously validated measures [34,35], and four studies used only previously validated measures [38,41,42,43,44].

The outcomes related to parent experiences or satisfaction with FCC interventions were the most common. Four studies [29,33,34,38] used “top-box” scores, which measure the highest possible responses to Likert-type survey items, such as “always” or “very good”. Top-box measurements are reliable for measuring parents’ attitudes, opinions, and perceptions [55]. Across all the studies that quantitatively measured parent satisfaction [41,44] or parent experience of an FCC intervention [29,31,32,33,35,38], the results were neutral to highly positive, with two exceptions. Pereira et al. reported mixed results on a peer intervention where one of five participants (20%) rated the program negatively [39]. Seliner et al. (*n* = 14) found non-significant decreases in four of the five domains of parent satisfaction and qualitative data to corroborate that parents were disappointed in the care they received *after* the intervention (they rated the preadmission program highly) [44]. Safety outcomes for children, such as error rates and incidence of accidental extubation, were measured in two studies and were either improved or had no change [32,38]. We could not identify any outcome patterns based on country, inpatient setting, or parent involvement in developing the FCC interventions. We were not able to compare the effects of different FCC interventions because only two studies reported an effect size, and insufficient data were presented in the other studies to calculate effect sizes. Leland et al. [34] reported a medium effect on parents’ spiritual well-being for a PICU holding intervention, and Seliner et al. [44] reported a small effect size for one non-significant parent satisfaction finding after a surgery preadmission intervention.

#### 3.4.2. Qualitative and Mixed-Methods Synthesis

The qualitative studies (including qualitative strands of mixed-methods studies) included interviews and/or focus groups with parents in four studies [30,35,37,44] and data extracted from an EHR in one study [36]. Only two studies found common themes of communication among parents and healthcare providers [30,37], whereas all other themes were unique. The diversity of themes may be due to the different patient populations, FCC interventions, and qualitative methods. However, the qualitative themes aligned with the FCC core principles as follows: The themes relating to the emotional needs of the parents, meeting their social needs, and granting parents the right to be with their child were consistent with the FCC principles of respect and dignity [30,35,37]. The themes about communication and parents expecting coordinated and continuous information aligned with the principle of information sharing [30,37,44]. The principle of parent participation was exemplified by a theme of feeling well-prepared [44]. The themes of having limitations to parent presence, advocating for their child, and expecting to be a part of the healthcare team represented the principle of collaboration [35,44]. No themes emerged from the studies that diverged from the FCC core principles. The two mixed-methods studies had qualitative findings that supported each study’s respective quantitative findings [35,44].

## 4. Discussion

### 4.1. Summary of Key Findings

This review evaluated findings from 16 studies on 15 FCC interventions with 3801 parents of hospitalized children from six diverse countries. After careful analysis, we found that there were no reported harms from an FCC intervention, and studies consistently reported favorable experiences and some significant improvements over usual care across a wide range of outcomes. Although some studies reported no significant differences between interventions and usual care, only a few participants in two small studies reported any negative perceptions of the interventions. We found the strongest support for information sharing interventions that were associated with improvements in parent experience, knowledge, and decreased stress for mothers. The parents who participated in respect and dignity interventions demonstrated improved communication and knowledge. The parents in participation interventions expressed strong positivity about being present with their child and reported increased spiritual well-being, self-efficacy, and decreased anxiety, among other results. These findings are the results of research where over half the studies involved parents and families in the development and/or implementation of FCC interventions.

We were limited in synthesizing data by the heterogeneity of illnesses experienced by children, intervention types, outcomes measured, measurement tools, and reporting of results. The quality of studies was, on average, moderate, and weaknesses were frequently related to study design and risks to internal validity. Taken together, the studies suggest that FCC interventions can increase parent knowledge, satisfaction with care and with FCC, participation in caregiving and decision-making, partnership with the healthcare team, and parent well-being.

### 4.2. Gaps and Unanswered Questions in Pediatric FCC Research

Many gaps remain in pediatric FCC inpatient research regarding the populations of interest, the FCC principles addressed, study design, methods, and outcome measures, as discussed below.

#### 4.2.1. Sampling Gaps

Half of the studies in our review included both mothers and fathers, suggesting a slight advancement in knowledge about FCC in relation to parental roles compared to previous research [56]. However, fathers remain under-represented in FCC research. In our review, these eight studies included samples with between 18% and 24% male caregivers. The four studies that limited the sample to mothers were from countries outside the U.S. Four studies did not report the gender of the parent participants. No studies reported on gender identity other than male or female. We also found insufficient representation of historically marginalized populations in this current review. One notable exception was a qualitative study that evaluated an FCC inpatient navigation program for Spanish- and English-speaking parents of hospitalized low-income children of color [30]. Future studies testing FCC interventions should include diverse samples and methods to examine if the opportunity access, uptake, and outcomes of the interventions are equitably distributed and generalizable [57]. Further qualitative research is also needed to understand why and how interventions may or may not be effective for child and parent outcomes across diverse settings and populations.

#### 4.2.2. Study Design and Method Gaps

Similar to previous reviews [58], we found a limited number of effectiveness studies, many of which had small sample sizes (i.e., less than 40 participants). None of these studies were RCTs, nor did any conduct comparative effectiveness studies of FCC interventions, with the majority being pre–post design or using a poorly defined usual care control group. The quantitative descriptive studies tended to have samples of more than 40 participants. Less than half the quantitative studies used rigorous statistical analysis methods. The quasi-experimental studies tended to report more improvements in outcomes from the interventions compared to usual care, but this may reflect selection or publication biases or inadequate control of potential confounders. One-quarter of the studies were pilot studies, which limits the interpretation and generalizability of findings and may provide context to quality appraisal scores. The number of pilot studies suggests that the field of FCC research in pediatric inpatient settings is still nascent. Less than half the quantitative studies used rigorous statistical analyses.

The most robust design for pediatric FCC intervention studies is an RCT with active controls to determine efficacy. Although individual-level randomization may not be possible for FCC interventions in pediatric settings, rigorous trial designs, such as cluster randomization or other implementation science designs can advance the quality of the research on pediatric FCC [59], as it has been achieved in neonatal FCC [60]. Comparative effectiveness trials of FCC interventions are also needed to address gaps in knowledge about scale-up and sustainability.

#### 4.2.3. Outcome Measurement Gaps

Similar to previous research, our review found inconsistencies in the outcomes measured and limited use of previously validated instruments [61]. Global consensus on key outcomes and validated measures for FCC are urgently needed if the field is to advance [62].

#### 4.2.4. Advances in FCC Intervention Research

Despite the gaps and unanswered questions, this review found advancements in FCC research in the past five years. First, studies are now reporting a family-centered care approach to designing the intervention and conducting the research. Nine of the 16 studies reported on family collaboration, such as including families in survey development, implementation (focus groups and interviews), or intervention workshops. The parents’ engagement and commitment served to co-produce instruments, interventions, or pilot studies. The parents served as advisors or had a role as peer mentors in an intervention.

Another advancement is identifying several well-designed and conducted studies in this review that can serve as examples for future research. Three effectiveness studies evaluating FCRs [38], a nighttime communication intervention [33], and a parent-touch intervention [34] are noteworthy because they were multi-site and/or had large sample sizes. One quantitative descriptive study about FCRs [31] also had a large sample (200 participants). However, RCTs remain the gold standard for determining intervention effectiveness and are urgently needed to compare FCC interventions.

### 4.3. Strengths and Limitations of the Systematic Review

A strength of this systematic review includes the use of the FCC core principles as a conceptual framework to organize this study’s findings in accordance with the four core principles, clear inclusion and exclusion criteria, and rigorous search, analysis, and synthesis methods. The FCC core principles enabled us to perform a meaningful synthesis and comparison of study findings despite the need for more consistency in interventions, measures, and outcomes. A limitation of this study is that we excluded ten studies that were unavailable in English, so we may have missed important global evidence for pediatric FCC. We also limited our review to outcomes related to children or parents/primary caregivers in the hospitalized setting. Some of these studies [29,30,37,39] measured other outcomes of the FCC interventions, including healthcare provider outcomes or health service outcomes, and a systematic review of FCC intervention impact on those outcomes is warranted. Because our data extraction focused exclusively on child and parent outcomes, we may have missed important information that led to a lower quality appraisal than the entire study merited. We also modified the MMAT tool to have a more specific scoring system, which may have introduced bias.

### 4.4. Implications for Practice, Policy, and Research

Despite the ongoing interest and previous research on FCC interventions in pediatric inpatient care, there has been limited progress in the past five years. While the historical evidence for FCC approaches remains strong, investment in updating the evidence for current healthcare contexts is needed. While RCTs may be prohibitively costly, rigorous quality-improvement studies, with consistent metrics, may offer more cost-effective ways to advance the practice of FCC [8]. Policymakers can also support the advancement of the field by including requirements for common metrics, regular audits, and the reporting of results for more dimensions of FCC.

Our findings raise an important question about how future studies should develop, measure, and compare pediatric FCC interventions to other interventions or usual care that is more provider- or hospital-centered. Because pediatric hospital care varies widely across inpatient settings and child diagnoses, interventions may need to be tailored, and the outcomes of interest may vary. Determining the best approach to increasing FCC interventions for improving the health outcomes of children and families is best achieved through local partnerships among clinicians, researchers, and families. Perhaps pediatric research studies can benefit from approaches used in the larger body of NICU FCC intervention research [8]. We strongly recommend greater attention and resources for more well-designed quantitative, qualitative, and mixed-methods research on the effectiveness of FCC interventions in pediatric hospital settings.

## Figures and Tables

**Table 1:** Core principles of family-centered care [6].

Core Principle	Description
Respect and Dignity	Healthcare practitioners listen to and honor patient and family perspectives and choices. Patient and family knowledge, values, beliefs, and cultural backgrounds are incorporated into the planning and delivery of care.
Information Sharing	Healthcare practitioners communicate and share complete and unbiased information with patients and families in ways that are affirming and useful. Patients and families receive timely, complete, and accurate information to effectively participate in care and decision-making.
Participation	Patients and families are encouraged and supported in participating in care and decision-making at the level they choose.
Collaboration	Patients, families, health care practitioners, and health care leaders collaborate in policy and program development, implementation, and evaluation; in facility design; in professional education; and in research, as well as in the delivery of care.

**Table 2:** Inclusion and exclusion criteria.

Inclusion Criteria:	Exclusion Criteria:
Study designs: Quantitative, i.e., experimental (RCT, pre-post), observational (comparative, descriptive, retrospective); mixed methods; qualitative, peer-reviewed journals.	Study designs: Systematic review, other review, commentary, case study of only one, concept analyses, policy statements, opinion pieces, studies from non-peer-reviewed journals. Gray literature was used for reference combing, but data were not extracted for synthesis.
Population: Children, premature through age 18, and their parents/primary caregivers (*neonatal populations were later excluded). We use the term “parent” to include mothers, fathers, or other primary caregivers.	Population: Studies that do not include hospitalized children or their families.
Setting: Hospitalized/inpatient	Setting: Studies in ambulatory or primary care settings, community settings.
Intervention: FCC interventions, programs or models that include one or more components: physical or psychological support for parents, communication with parents, education, partnership, shared decision-making, or parent participation in care. Must promote core principles of FCC of dignity and respect, information sharing, participation, collaboration, and negotiation.	Intervention: Studies that do not describe or evaluate an FCC model, approach, or intervention. Studies that involve only one specific technique or only the physical environment.
Comparison: Other models of care or interventions that do not explicitly include FCC interventions (for studies with a comparison group). Not applicable to qualitative research.	Comparison: No exclusions.
Outcomes: Parent/family outcomes: knowledge or understanding, physical or psychological health, satisfaction, attitudes, behavior, interaction with child, adverse events for child or parent. Child outcomes: physical, psychological, developmental, satisfaction, attitudes, behavior.	Outcomes: Staff outcomes/experiences, health service outcomes.
Language: Published in English.	Language: Any language other than English.
Publication date: 1 January 2017 to 6 February 2024	Publication date: Prior to 1 January 2017, or after 6 February 2024, or research published in 2017 or 2018 and included in a prior systematic review.

**Table 3:** Outcome measures and psychometric properties or development strategies.

Name	Outcomes/Measurement Method	Developed for the Study (Yes/No)	Constructs (Number of Items). Psychometric Properties. Measurement development Notes.
Chamblee and Miles (2021) [29]	CLABSI knowledge. Parent perception of FCC. Online survey.	Yes	CLABSI knowledge (4 items); FCC (14 items). Face validity by FCC expert. Internal validity on 1062 surveys, Cronbach’s alpha 0.71. Developed with input from parent advisors.
Chisolm et al., (2022) [30]	Qualitative Parent perceptions of participation in the navigation program. Interviews	Yes	Interview guide available “from the author on request”.
Glick et al. (2020) [31]	Parent perspectives on FCRs. Parent resource preference. Survey read aloud by researcher.	Yes	Participation, understanding, preferences (10 items) Developed with input from parent advisors. Survey published in appendix.
Kelly et al. (2017) [32]	Parent use of portal. Parent perceptions. Online survey.	Yes	Perception of portal ease of use, usefulness, satisfaction, impact on participation, communication, errors, care quality (number of items not specified; 3 open-ended). Adapted from several published surveys. Pilot-tested survey with a multidisciplinary team including parents. Supplementary link to survey was inactive.
Khan et al. (2017) [33]	Parent experience with FCC. Nighttime intervention survey. Shared understanding between parent and staff survey. Paper survey.	Yes	23 items; Closed and open-ended questions. Used a survey methodologist. Cognitively pilot tested with healthcare team. Survey construction involved parents.
Khan et al. (2018) [38]	Family experience survey. Available in paper, verbal or electronic formats. Survey took 5–10 min to complete. Medical errors: harmful and non-harmful. Communication: Observation.	Yes	Family experience (25 items). Cognitively tested, pilot tested. Reading level determined, translated to 4 languages. Error data extracted from EHR. Iteratively developed and pilot tested a structured observation tool using audio recordings from FCR.
Krisnana et al. (2019) [42]	Parent stress. DASS-21 scale. Survey.	No	DASS scale (21 items), a previously validated and translated survey [45]. Reliability coefficient compared with with the Beck Depression and Anxiety Inventories = 0.9483.
Leland et al. (2017) [34]	Caregiver spiritual well-being. FACIT -Sp-12 Survey. Caregiver perceptions of care. Survey. Parents completed surveys on day 1 (day of enrollment) and day 4. Unplanned extubations: Monitored by standard procedures.	Both previously validated and author-developed	FACIT-Sp-12 (12 items) [46], modified the previously validated survey with permission. Caregiver perceptions (4 items) Author developed based on FCC principles. Unplanned extubation rate: number of events per 100 ventilator days.
Nankali et al. (2023) [43]	Practices and skills of mothers. Checklist of care completed by 1 of 2 researchers.	No	AGE (56 items), adapted the previously published checklist [47]. Cronbach’s alpha = 0.89. Content validity established with nurses, pediatricians, and faculty. CVI = 0.72–0.93 for each item. Cohen’s kappa = 0.77, 95% CI (0.62–0.85).
O’Connell et al. (2017) [35]	Experience of caregivers who were present. Experience of caregivers who were not present. Surveys administered in telephone interviews.	Both previously validated and author-developed	Family presence during invasive procedures survey used in prior research (36 items) [48]. Cronbach’s alpha = 0.92 and 0.81 in prior research; 0.89 in O’Connell et al. (2017). Construct validity with exploratory factor analysis yielded 2 subscales. Family not present survey (17 items). Developed using the Lynn procedure [49]. Cronbach’s alpha = 0.89. Interview script not provided.
Pereira et al. (2021) [39]	Family volunteer survey. Family peer mentor survey. Surveys available either electronically or on paper.	Yes	Developed with input from health care team and parent advisors.
Pollock et al. (2022) [36]	Qualitative. Data extracted from EHR.	Yes	A systematic retrospective analysis of EHR notes written by the family partners who made visits with parents.
Runaas et al. (2017) [37]	Qualitative. Interviews.	Yes	Interview script provided in supplementary materials.
Seliner et al. (2017) [44]	Parental satisfaction with FCC. (MPOC-20) survey.	No	MPOC-20 (20 items), a well-validated instrument [50]. Previous studies reported Cronbach’s alpha = 0.63–0.92. Interview script with example questions reported.
Uhm and Choi (2019) [40]	Parents’ desired information, preferred type of care, and participation. Verbal questioning by nurses. Length of participation. Physical engagement. Psychological connectedness. Nurse observation.	Yes	Open-ended questions. Content validity established by multidisciplinary team, including parents. CVI = 0.96–1.0 for each item. Observation of time and type of parent participation. Physical engagement and psychological connectedness were rated on visual analog scales according to nurses’ observations.
Um and Kim (2019) [41]	Parent satisfaction in the PICU. Parents’ perceived self-efficacy. Parenting confidence. Perceived partnership. Parent–nurse partnership. Maternal anxiety. Infant outcomes: Time to reach full feed. LOS.	No	EMPATH PICU Parental Satisfaction Scale (30 items) [51]. Cronbach’s alpha = 0.97. Parenting Confidence Scale (15 items) [52]. Cronbach’s alpha = 0.90. Parent–Nurse Partnership Scale (34 items) [53]. Cronbach’s alpha = 0.9. Speilberger State Anxiety Scale, Korean version (20 items) [54]. Cronbach’s alpha = 0.95. Time to reach infant feeding goal of 100 mL/kg/day. LOS, days of postoperative hospitalization.

AGE, acute gastroenteritis; CI, confidence interval; CLABSI, central line-associated bloodstream infection; CVI, content validity index; DASS, depression and anxiety stress scale; EHR, electronic health record; FACIT-SP12, Functional assessment of chronic illness therapy–spiritual well-being; FCC, family-centered care; FCRs, family-centered rounds; EHR, electronic health record; LOS, length of stay; MPOC-20, measurement of processes of care; PICU, pediatric intensive care unit.

## Data Availability

The original contributions presented in this study are included in the article/supplementary material; the data are available on request from the corresponding author.

## Supplementary Materials

The following supporting information can be downloaded at: https://www.mdpi.com/article/10.3390/children11080949/s1, S1: Table of PRISMA2020 Checklist and Abstract Checklist. S2: Table of Search Strategy and Search Terms. S3: Table of Modified MMAT Scoring Search Strategy. S4: Table of Study Characteristics, FCC Interventions, and Results.

## References

[1] Epstein R.M., Fiscella K., Lesser C.S., Stange K.C. (2010). Why the nation needs a policy push on patient-centered health care. Health Aff..

[2] AAP Publications (2018). AAP Publications Reaffirmed or Retired. Pediatrics.

[3] Mueller B.U., Neuspiel D.R., Fisher E.R.S., Franklin W., Adirim T., Bundy D.G., Ferguson L.E., Gleeson S.P., Leu M., Council on Quality Improvement and Patient Safety, Committee on Hospital Care (2019). Principles of pediatric patient safety: Reducing harm due to medical care. Pediatrics.

[4] Tripodi M., Siano M.A., Mandato C., De Anseris A.G.E., Quitadamo P., Guercio Nuzio S., Siani P., Vajro P. (2019). Humanization interventions in general pediatric wards: A systematic review. Eur. J. Pediatr..

[5] Fernandes A.K., Wilson S., Nalin A.P., Philip A., Gruber L., Kwizera E., Sydelko B.S., Forbis S.G., Lauden S. (2021). Pediatric family-centered rounds and humanism: A systematic review and qualitative meta-analysis. Hosp. Pediatr..

[6] IPFCC Institute for Patient and Family-Centered Care. https://www.ipfcc.org/.

[7] Kokorelias K.M., Gignac M.A.M., Naglie G., Cameron J.I. (2019). Towards a universal model of family centered care: A scoping review. BMC Health Serv. Res..

[8] Franck L.S., Axelin A., Van Veenendaal N.R., Bacchini F. (2023). Improving Neonatal Intensive Care Unit Quality and Safety with Family-Centered Care. Clin. Perinatol..

[9] Duke T. (2018). New WHO standards for improving the quality of healthcare for children and adolescents. Arch. Dis. Child..

[10] Phiri P.G.M.C., Chan C.W.H., Wong C.L. (2020). The scope of family-centered care practices, and the facilitators and barriers to implementation of family-centered care for hospitalized children and their families in developing countries: An integrative review. J. Pediatr. Nurs..

[11] Segers E., Ockhuijsen H., Baarendse P., van Eerden I., van den Hoogen A. (2019). The impact of family centered care interventions in a neonatal or paediatric intensive care unit on parents’ satisfaction and length of stay: A systematic review. Intensive Crit. Care Nurs..

[12] Ladak L.A., Premji S.S., Amanullah M.M., Haque A., Ajani K., Siddiqui F.J. (2013). Family-centered rounds in Pakistani pediatric intensive care settings: Non-randomized pre- and post-study design. Int. J. Nurs. Stud..

[13] Lester B.M., Salisbury A.L., Hawes K., Dansereau L.M., Bigsby R., Laptook A., Taub M., Lagasse L.L., Vohr B.R., Padbury J.F. (2016). 18-Month Follow-Up of Infants Cared for in a Single-Family Room Neonatal Intensive Care Unit. J. Pediatr..

[14] Melnyk B.M., Feinstein N.F., Alpert-Gillis L., Fairbanks E., Crean H.F., Sinkin R.A., Stone P.W., Small L., Tu X., Gross S.J. (2006). Reducing premature infants’ length of stay and improving parents’ mental health outcomes with the Creating Opportunities for Parent Empowerment (COPE) Neonatal Intensive Care Unit Program: A randomized, controlled trial. Pediatrics.

[15] Ortenstrand A., Westrup B., Brostrom E.B., Sarman I., Akerstrom S., Brune T., Lindberg L., Waldenstrom U. (2010). The Stockholm Neonatal Family Centered Care Study: Effects on Length of Stay and Infant Morbidity. Pediatrics.

[16] De Bernardo G., Svelto M., Giordano M., Sordino D., Riccitelli M. (2017). Supporting parents in taking care of their infants admitted to a neonatal intensive care unit: A prospective cohort pilot study. Ital. J. Pediatr..

[17] October T.W., Hinds P.S., Wang J., Dizon Z.B., Cheng Y.I., Roter D.L. (2016). Parent Satisfaction With Communication Is Associated With Physician’s Patient-Centered Communication Patterns During Family Conferences. Pediatr. Crit. Care Med..

[18] Penticuff J.H., Arheart K.L. (2005). Effectiveness of an Intervention to Improve Parent-Professional Collaboration in Neonatal Intensive Care. J. Perinat. Neonatal Nurs..

[19] Stevens D.C., Helseth C.C., Khan M.A., Munson D.P., Reid E.J. (2011). A Comparison of Parent Satisfaction in an Open-Bay and Single-Family Room Neonatal Intensive Care Unit. HERD.

[20] Trujillo J.A., Fernandez Y., Ghafoori L., Lok K., Valencia A. (2017). Interdisciplinary Family Conferences to Improve Patient Experience in the Neonatal Intensive Care Unit. Health Soc. Work.

[21] Voos K.C., Ross G., Ward M.J., Yohay A.-L., Osorio S.N., Perlman J.M. (2011). Effects of implementing family-centered rounds (FCRs) in a neonatal intensive care unit (NICU). J. Matern. Fetal Neonatal Med..

[22] Weiss S., Goldlust E., Vaucher Y.E. (2010). Improving parent satisfaction: An intervention to increase neonatal parent-provider communication. J. Perinatol..

[23] Al-Momani M.M. (2011). Mothers’ Satisfaction Towards Pediatric Nursing Care. Middle East J. Nurs..

[24] Page M.J., McKenzie J.E., Bossuyt P.M., Boutron I., Hoffmann T.C., Mulrow C.D., Shamseer L., Tetzlaff J.M., Akl E.A., Brennan S.E. (2021). The PRISMA 2020 statement: An updated guideline for reporting systematic reviews. BMJ.

[25] Covidence Systematic Review Software. Veritas Health Innovation, Melbourne, Australia. https://www.covidence.org.

[26] Hong Q.N., Fàbregues S., Bartlett G., Boardman F., Cargo M., Dagenais P., Gagnon M.-P., Griffiths F., Nicolau B., O’Cathain A. (2018). The Mixed Methods Appraisal Tool (MMAT) version 2018 for information professionals and researchers. Educ. Inf..

[27] Hong Q.N., Gonzalez-Reyes A., Pluye P. (2018). Improving the usefulness of a tool for appraising the quality of qualitative, quantitative and mixed methods studies, the Mixed Methods Appraisal Tool (MMAT). J. Eval. Clin. Pract..

[28] Brunton G., Oliver S., Thomas J. (2020). Innovations in framework synthesis as a systematic review method. Res. Synth. Methods.

[29] Chamblee T., Miles D. (2021). A Prospective Study of Family Engagement for Prevention of Central Line-associated Blood Stream Infections. Pediatr. Qual. Saf..

[30] Chisholm H., Kershaw T., Guerra L.S., Bocek K., Garcia Y., Lion K.C. (2022). A Realist Evaluation Analysis of a Novel Multi-Faceted Inpatient Patient Navigation Program. Acad. Pediatr..

[31] Glick A.F., Goonan M., Sherman J., Sandmeyer D., Gold-von Simson G. (2020). Parent perspectives on participation in family-centered rounds and informaiontal resources use. Front. Pediatr..

[32] Kelly M.M., Hoonakker P.L., Dean S.M. (2017). Using an inpatient portal to engage families in pediatric hospital care. J. Am. Med. Inf. Assoc..

[33] Khan A., Baird J., Rogers J.E., Furtak S.L., Williams K.A., Allair B., Litterer K.P., Sharma M., Smith A., Schuster M.A. (2017). Parent and Provider Experience and Shared Understanding After a Family-Centered Nighttime Communication Intervention. Acad. Pediatr..

[34] Leland B.D., Nitu M.E., Hancock M., Moody K., Gunderman R., Moser E., Rowan C.M. (2017). Prospective Evaluation of Physical Contact with Critically Ill Child on Caregiver Spiritual Wellbeing. J. Pediatr..

[35] O’Connell K., Fritzeen J., Guzzetta C.E., Clark A.P., Lloyd C., Scott S.H., Aldridge M.D., Kreling B. (2017). Family Presence During Trauma Resuscitation: Family Members’ Attitudes, Behaviors, and Experiences. Am. J. Crit Care Off. Publ. Am. Assoc. Crit-Care Nurses.

[36] Pollock M.D., Ming D., Chung R.J., Maslow G. (2022). Parent-to-parent peer support for children and youth with special health care needs: Preliminary evaluation of a family partner program in a healthcare system. J. Pediatr. Nurs..

[37] Runaas L., Hanauer D., Maher M., Bischoff E., Fauer A., Hoang T., Munaco A., Sankaran R., Gupta R., Seyedsalehi S. (2017). BMT Roadmap: A User-Centered Design Health Information Technology Tool to Promote Patient-Centered Care in Pediatric Hematopoietic Cell Transplantation. Biol. Blood Marrow. Transpl..

[38] Khan A., Spector N.D., Baird J.D., Ashland M., Starmer A.J., Rosenbluth G., Garcia B.M., Litterer K.P., E Rogers J., Dalal A.K. (2018). Patient safety after implementation of a coproduced family centered communication programme: Multicenter before and after intervention study. BMJ.

[39] Pereira N., MacDonald C., Drobot A., Bennett A., Ali A.-B., Garros D. (2021). A peer and volunteer program for patients and their families in the pediatric intensive care unit: A pilot program evaluation. Front. Pediatr..

[40] Uhm J.-Y., Choi M.-Y. (2019). Mothers’ needs regarding partnerships with nurses during care of infants with congenital heart defects in a paediatric cardiac intensive care unit. Intensive Crit. Care Nurs..

[41] Uhm J.-Y., Kim H.S. (2019). Impact of the mother–nurse partnership programme on mother and infant outcomes in paediatric cardiac intensive care unit. Intensive Crit. Care Nurs..

[42] Krisnana I., Sulistyarini H., Rachmawati P.D., Arief Y.S., Kurnia I.D. (2019). Reducing acute stess disorders in mothers of leukemic children by means of the family centered empowerment module (FACE). Cent. Eur. J. Nurs. Midwifery.

[43] Nankali H., Cheraghi F., Tehrani T.H., Mohammadi Y., Azadimoghtader M. (2023). Mothers participation in caring for hospitalized children with acute gastroenteritis: A quasi-experimental study. Nurs. Open.

[44] Seliner B., Latal B., Spirig R. (2017). Effectiveness of a nurse-led preadmission intervention for parents of children with profound multiple disabilities undergoing hip-joint surgery: A quasi-experimental pilot study. J. Spec. Pediatr. Nurs..

[45] Lovibond P.F., Lovibond S.H. (1995). The structure of negative emotional states: Comparison of the Depression Anxiety Stress Scales (DASS) with the Beck Depression and Anxiety Inventories. Behav. Res. Ther..

[46] Allen D., Marshall E.S. (2010). Spirituality as a coping resource for African American parents of chronically ill children. MCN Am. J. Matern Child. Nurs..

[47] van den Berg J., Berger M.Y. (2011). Guidelines on acute gastroenteritis in children: A critical appraisal of their quality and applicability in primary care. BMC Fam. Pract..

[48] Meyers T.A., Eichhorn D.J., Guzzetta C.E., Clark A.P., Klein J.D., Taliaferro E., Calvin A. (2004). Family Presence During Invasive Procedures and Resuscitation: The Experience of Family Members, Nurses, and Physicians. Top Emerg. Med..

[49] Lynn M.R. (1986). Determination and quantification of content validity. Nurs. Res..

[50] Cunningham B.J., Rosenbaum P.L. (2014). Measure of Processes of Care: A review of 20 years of research. Dev. Med. Child Neurol..

[51] Latour J.M., Duivenvoorden H.J., Tibboel D., Hazelzet J.A. (2013). The shortened EMpowerment of PArents in THe Intensive Care 30 questionnaire adequately measured parent satisfaction in pediatric intensive care units. J. Clin. Epidemiol..

[52] Črnčec R., Barnett B., Matthey S. (2008). Development of an instrument to assess perceived self-efficacy in the parents of infants. Res. Nurs. Health.

[53] Choi M.Y., Bang K.-S. (2013). Development and testing of a pediatric nurse parent partnership scale. J. Korean Acad. Nurs..

[54] Kim J.T., Shin D.K. (1978). A study based on the standardization of the STAI for Korea. New Med. J..

[55] HCAHPS Hospital Consumer Assessment of Healthcare Providers and Systems: Tables on HCAHPS On-Line. https://hcahpsonline.org/en/summary-analyses/.

[56] Benzies K.M., E Magill-Evans J., Hayden K., Ballantyne M. (2013). Key components of early intervention programs for preterm infants and their parents: A systematic review and meta-analysis. BMC Pregnancy Childbirth.

[57] Morgan P.L., Hu E.H., Woods A.D., Gloski C.A., Wang Y. (2023). Disparities in Family-Centered Care Among US Children and Youth with Special Healthcare Needs. J. Pediatr..

[58] Davidson J.E., Aslakson R.A., Long A.C., Puntillo K.A., Kross E.K., Hart J., Cox C.E., Wunsch H., Wickline M.A., Nunnally M.E. (2017). Guidelines for Family-Centered Care in the Neonatal, Pediatric, and Adult ICU. Crit. Care Med..

[59] Toolkit Part 1: Implementation Science Methodologies and Frameworks—Fogarty International Center @ NIH. Fogarty International Center. https://www.fic.nih.gov:443/About/center-global-health-studies/neuroscience-implementation-toolkit/Pages/methodologies-frameworks.aspx.

[60] O’Brien K., Robson K., Bracht M., Cruz M., Lui K., Alvaro R., da Silva O., Monterrosa L., Narvey M., Ng E. (2018). Effectiveness of Family Integrated Care in neonatal intensive care units on infant and parent outcomes: A multicentre, multinational, cluster-randomised controlled trial. Lancet Child Adolesc. Health.

[61] Curley M., Hunsberger M., Harris S. (2013). Psychometric Evaluation of the Family-Centered Care Scale for Pediatric Acute Care Nursing. Nurs. Res..

[62] Al-Motlaq M.A., Carter B., Neill S., Hallstrom I.K., Foster M., Coyne I., Arabiat D., Darbyshire P., Feeg V.D., Shields L. (2019). Toward developing consensus on family-centred care: An international descriptive study and discussion. J. Child Health Care.

