# Supplementary material for: Child and Family Outcomes and Experiences Related to Family-Centered Care Interventions for Hospitalized Pediatric Patients: A Systematic Review
Source: Children (Basel). 2024 Aug 6;11(8):949. doi: 10.3390/children11080949 (PMC11353055; doi:10.3390/children11080949)
Supplement: Supplementary file 1 [file children-11-00949-s001.zip › children-3108184-supplementary.pdf]

Supplementary Materials

S1. PRISMA 2020 Checklist and Abstract Checklist

Section and Topic	Item #	Checklist item	Location where item is reported
**TITLE**			
Title	1	Identify the report as a systematic review.	Title page
**ABSTRACT**			
Abstract	2	See the PRISMA 2020 for Abstracts checklist.	See Abstract check-list below
**INTRODUCTION**			
Rationale	3	Describe the rational for the review in the context of existing knowledge.	Pages 1-3
Objectives	4	Provide an explicit statement of the objective(s) or question(s) the review addresses.	Page 3
**METHODS**			
Eligibility crite-ria	5	Specify the inclusion and exclusion criteria for the review and how studies were grouped for the syn-theses.	Page 4-5
Information sources	6	Specify all databases, registers, websites, organisations, reference lists and other sources searched or consulted to identify studies. Specify the date when each source was last searched or consulted.	Page 4, Appendix A1
Search strategy	7	Present the full search strategies for all databases, registers and websites, including any filters and lim-its used.	Appendix A1
Selection pro-cess	8	Specify the methods used to decide whether a study met the inclusion criteria of the review, including how many reviewers screened each record and each report retrieved, whether they worked inde-pendently, and if applicable, details of automation tools used in the process.	Pages 5
Data collection process	9	Specify the methods used to collect data from reports, including how many reviewers collected data from each report, whether they worked independently, any processes for obtaining or confirming data from study investigators, and if applicable, details of automation tools used in the process.	Page 5
Data items	10a	List and define all outcomes for which data were sought. Specify whether all results that were	Pages 5. Appendix

Section and Topic	Item #	Checklist item	Location where item is reported
		compatible with each outcome domain in each study were sought (e.g. for all measures, time points, analyses), and if not, the methods used to decide which results to collect.	A3
	10b	List and define all other variables for which data were sought (e.g. participant and intervention characteristics, funding sources). Describe any assumptions made about any missing or unclear information.	Pages 5-7, Appendix A3
Study risk of bias assessment	11	Specify the methods used to assess risk of bias in the included studies, including details of the tool(s) used, how many reviewers assessed each study and whether they worked independently, and if applicable, details of automation tools used in the process.	Page 5-7
Effect measures	12	Specify for each outcome the effect measure(s) (e.g. risk ratio, mean difference) used in the synthesis or presentation of results.	Page 7
Synthesis methods	13a	Describe the processes used to decide which studies were eligible for each synthesis (e.g. tabulating the study intervention characteristics and comparing against the planned groups for each synthesis (item #5)).	Page 7
	13b	Describe any methods required to prepare the data for presentation or synthesis, such as handling of missing summary statistics, or data conversions.	N/A
	13c	Describe any methods used to tabulate or visually display results of individual studies and syntheses.	Page 7, Appendix A3
	13d	Describe any methods used to synthesize results and provide a rationale for the choice(s). If meta-analysis was performed, describe the model(s), method(s) to identify the presence and extent of statistical heterogeneity, and software package(s) used.	Page 7
	13e	Describe any methods used to explore possible causes of heterogeneity among study results (e.g. subgroup analysis, meta-regression).	N/A
	13f	Describe any sensitivity analyses conducted to assess robustness of the synthesized results.	N/A
Reporting bias assessment	14	Describe any methods used to assess risk of bias due to missing results in a synthesis (arising from reporting biases).	Page 7

Section and Topic	Item #	Checklist item	Location where item is reported
Certainty assessment	15	Describe any methods used to assess certainty (or confidence) in the body of evidence for an outcome.	N/A
**RESULTS**			
Study selection	16a	Describe the results of the search and selection process, from the number of records identified in the search to the number of studies included in the review, ideally using a flow diagram.	Pages 6, 7
	16b	Cite studies that might appear to meet the inclusion criteria, but which were excluded, and explain why they were excluded.	Pages 5,6
Study characteristics	17	Cite each included study and present its characteristics.	Pages 8-17, Appendix A3
Risk of bias in studies	18	Present assessments of risk of bias for each included study.	Page 11-2, Appendix 2
Results of individual studies	19	For all outcomes, present, for each study: (a) summary statistics for each group (where appropriate) and (b) an effect estimate and its precision (e.g. confidence/credible interval), ideally using structured tables or plots.	Pages 8-17, Appendix 3
Results of syntheses	20a	For each synthesis, briefly summarise the characteristics and risk of bias among contributing studies.	Page 17
	20b	Present results of all statistical syntheses conducted. If meta-analysis was done, present for each the summary estimate and its precision (e.g. confidence/credible interval) and measures of statistical heterogeneity. If comparing groups, describe the direction of the effect.	N/A
	20c	Present results of all investigations of possible causes of heterogeneity among study results.	N/A
	20d	Present results of all sensitivity analyses conducted to assess the robustness of the synthesized results.	N/A
Reporting biases	21	Present assessments of risk of bias due to missing results (arising from reporting biases) for each synthesis assessed.	N/A
Certainty of evidence	22	Present assessments of certainty (or confidence) in the body of evidence for each outcome assessed.	N/A
**DISCUSSION**			

Section and Topic	Item #	Checklist item	Location where item is reported
Discussion	23a	Provide a general interpretation of the results in the context of other evidence.	Pages 18-19
	23b	Discuss any limitations of the evidence included in the review.	Page 19
	23c	Discuss any limitations of the review processes used.	Page 19-20
	23d	Discuss implications of the results for practice, policy, and future research.	Page 20
**OTHER INFORMATION**			
Registration and protocol	24a	Provide registration information for the review, including register name and registration number, or state that the review was not registered.	Review not registered
	24b	Indicate where the review protocol can be accessed, or state that a protocol was not prepared.	Protocol not prepared
	24c	Describe and explain any amendments to information provided at registration or in the protocol.	N/A
Support	25	Describe sources of financial or non-financial support for the review, and the role of the funders or sponsors in the review.	Page 20
Competing interests	26	Declare any competing interests of review authors.	Page 20
Availability of data, code and other materials	27	Report which of the following are publicly available and where they can be found: template data collection forms; data extracted from included studies; data used for all analyses; analytic code; any other materials used in the review.	Page 20

From: *From:* Page MJ, McKenzie JE, Bossuyt PM, Boutron I, Hoffmann TC, Mulrow CD, et al. The PRISMA 2020 statement: an updated guideline for reporting systematic reviews. Syst Rev 10, 89 (2021). https://doi.org/10.1186/s13643-021-01626-4

Item #	Checklist item for ABSTRACTS	Reported (Yes/No)
**TITLE**		
1	Identify the report as a systematic review.	Y

Item #	Checklist item for ABSTRACTS	Reported (Yes/No)
**BACKGROUND**		
2	Provide an explicit statement of the main objective(s) or question(s) the review addresses.	Y
**METHODS**		
3	Specify the inclusion and exclusion criteria for the review.	Acknowledged criteria but limited by word count
4	Specify the information sources (e.g. databases, registers) used to identify studies and the date when each was last searched.	Y
5	Specify the methods used to assess risk of bias in the included studies.	Y
6	Specify the methods used to present and synthesise results.	Y
**RESULTS**		
7	Give the total number of included studies and participants and summarise relevant characteristics of studies.	Number stated, participants and characteristics limited by word count
8	Present results for main outcomes, preferably indicating the number of included studies and participants for each. If meta-analysis was done, report the summary estimate and confidence/credible interval. If comparing groups, indicate the direction of the effect (i.e. which group is favoured).	Y
**DISCUSSION**		
9	Provide a brief summary of the limitations of the evidence included in the review (e.g. study risk of bias, inconsistency and imprecision).	Y
10	Provide a general interpretation of the results and important implications.	Y
**OTHER**		
11	Specify the primary source of funding for the review.	N (reported in manuscript)
12	Provide the register name and registration number.	N/A

From: Page MJ, McKenzie JE, Bossuyt PM, Boutron I, Hoffmann TC, Mulrow CD, et al. The PRISMA 2020 statement: an updated guideline for reporting systematic reviews. Syst Rev 10, 89 (2021). https://doi.org/10.1186/s13643-021-01626-4

S2. Search Strategy and Search Terms

Family-centered care

Date searched: 2/13/2023

Date search updated: 2/6/2024

<u>PubMed</u>:

(“family-centered care” OR "Family Nursing"[Mesh] OR “family nursing” OR “family integrated care” OR “Patient-Centered Care”[Mesh] OR “patient-centered care” OR “patient-centered nursing”) AND (“Child”[Mesh] OR child OR children OR “Adolescent”[Mesh] OR adolescent* OR teen* OR “Infant”[Mesh] OR infant* OR neonat* OR newborn OR pediatric) AND ("Inpatients"[Mesh] OR inpatient* OR hospitalized* OR “hospital-based” OR NICU OR “newborn intensive care” OR "Intensive Care Units, Neonatal"[Mesh] OR “pediatric ICU” OR “pediatric intensive care” OR "Intensive Care Units, Pediatric"[Mesh])

References = 1,178

<u>Web of Science</u>:

(“family-centered care” OR “family nursing” OR “family integrated care” OR “patient-centered care” OR “patient-centered nursing”) AND (child OR children OR adolescent* OR teen* OR infant* OR neonat* OR newborn OR pediatric) AND (inpatient* OR hospitalized* OR “hospital-based” OR NICU OR “newborn intensive care” OR “pediatric ICU” OR “pediatric intensive care”)

References = 773

<u>Embase (limit to articles/articles in press):</u>

('family centered care'/exp OR 'family centered care' OR 'family integrated care'/exp OR 'family integrated care') AND ('pediatric'/exp OR 'pediatric' OR 'newborn'/exp OR 'newborn' OR 'infant'/exp OR 'infant' OR 'adolescent'/exp OR 'adolescent') AND ('hospital patient'/exp OR 'hospital patient' OR 'intensive care unit'/exp OR 'intensive care unit' OR hospitalized OR 'hospitalized child'/exp OR 'hospitalized child' OR 'hospitalized adolescent'/exp OR 'hospitalized adolescent' OR 'hospitalized infant'/exp OR 'hospitalized infant') AND ([article]/lim OR [article in press]/lim)

References = 477

<u>CINAHL (limit to scholarly articles)</u>:

(“family-centered care” OR “family nursing” OR “family integrated care” OR “patient-centered care” OR “patient-centered nursing”) AND (child OR children OR adolescent* OR teen* OR infant* OR neonat* OR newborn OR pediatric) AND (inpatient* OR hospitalized* OR “hospital-based” OR NICU OR “newborn intensive care” OR “pediatric ICU” OR “pediatric intensive care”)

References = 1,507

PsycINFO:

("family-centered care" OR "family nursing" OR "family integrated care" OR "patient-centered care" OR "patient-centered nursing") AND (child OR children OR adolescent* OR teen* OR infant* OR neonat* OR newborn OR pediatric) AND (inpatient* OR hospitalized* OR "hospital-based" OR NICU OR "newborn intensive care" OR "pediatric ICU" OR "pediatric intensive care")

References =113

S3. Modified MMAT Scoring

A. Design: Quasi-experimental

First Author	3.1.a. Clearly reports inclusion and exclusion criteria	3.1.b. States why some chose not to partici-pate	3.1.c. Attempts to repre-sent the target popula-tion	3.2.a. Measure-ment variables are clearly defined	3.2.b. Variables are accurate-ly meas-ured	3.2.c. Measure-ments are justified and appropri-ate	3.2.d. Uses Validated and reliability tested measures	3.3. Outcome data are at least 80% complete	3.4.a. Collected data on confound-ers	3.4.b. Analyzed data on confound-ers	3.5. Inter-vention was adminis-tered as intended	Total/ total possible	%
Chamblee[29]	1	0	0	1	1	1	1	1	0	0	1	7/11	64%
Khan[33]	1	0	1	1	1	1	1	0	1	1	0	8/11	73%
Khan[38]	1	0	1	1	1	1	1	0	1	1	1	9/11	82%
Krisnana[42]	1	0	0	1	1	0	1	0	1	0	1	6/11	55%
Leland[34]	1	0	0	1	1	1	1	0	1	0	1	7/11	64%
Nankali[43]	1	0	0	0	1	1	1	1	1	1	1	8/11	73%
O'Connell[35] (MM)	1	0	0	1	1	1	1	1	1	0	1	8/11	73%
Pereira[39]	1	0	0	1	1	1	0	0	0	0	0	4/11	36%
Seliner[44] (MM)	1	1	0	1	1	1	1	1	1	0	0	8/11	73%
Uhm[41]	1	0	0	1	1	1	1	1	1	1	1	9/11	82%

B. Design: Quantitative Descriptive

Author	4.1.a. Relevant sample source	4.1.b. Clear justification of sampling framework	4.1.c. Adequate sampling procedure	4.2.a. Clearly reports inclusion and exclusion criteria	4.2.b. States why some chose not to participate	4.2.c. Attempts to achieve a representative sample	4.3.a. Variables clearly defined	4.3.b. Variables accurately measured	4.3.c. Measures justified and appropriate	4.3.d. Measures validated and reliability tested
Glick[31]	1	1	1	1	0	0	1	1	1	1
Kelly[32]	1	1	1	1	0	1	1	1	1	1
Uhm[40]	1	0	1	1	0	0	1	1	1	1
Author *continued*	4.3.e. Questionnaires pre-tested prior to data collection	4.4.a Has a low non-response rate	4.4.b Reasons given for non-response	4.4.c. Statistical compensation for non-response	4.5.a. Clearly stated statistical analysis	4.5.b. Justified statistical analysis for research question			Total of Total Possible	%
Glick[31]	1	1	1	1	1	0			13/16	81%
Kelly[32]	1	0	0	0	1	0			11/16	69%
Uhm [40]	1	0	0	0	1	1			10/16	63%

A. Design: Qualitative

Author	1.1 Appropriate qualitative approach for the research question	1.2.a. Adequate method of data collection	1.2.b. Adequate form of data and justification for any changes	1.3 Findings adequately derived from data analysis	1.4 Interpretation of results substantiated by data	1.5 Data sources, collection, analysis, and interpretation are linked	Total of total possible	Total %
Chisholm[30]	1	1	1	1	1	1	6/6	100%
O'Connell[35]	1	1	1	0	1	1	5/6	83%
Pollock[36]	1	0	1	1	1	0	4/6	67%
Runaas[37]	0	1	0	1	1	0	3/6	50%
Seliner[44]	1	1	0	1	1	1	5/6	83%

MM: Mixed Methods

S4. Study Characteristics, FCC Interventions, and Results

Authors (Year), Country	Study Design/Methods, Quality Appraisal, Analyses	Setting, Sample, Family Involvement	Intervention Description, FCC Principles (Primary Emphasis*)	Quasi-Experimental Results*, Quantitative Results, Qualitative Results
Chamblee & Miles (2021)[29]<br><br>U.S.	Quasi-experimental comparing different groups pre and post using surveys<br><br>64%<br><br>Wilcoxon Rank Sum test, *t*-test, and Chi-square test.<br><br>Confounders were not accounted for in the design and analysis.	Parents of children in a PICU.<br><br>Pre, n = 59<br>Post, n = 62<br><br>Intervention was created with input from the Patient and FCC committee, which included patient/family representatives.	Intervention to prevent CLABSI included written information on basic prevention and concrete steps on how parents could partner with the medical team to prevent a CLABSI.<br><br>Respect and Dignity<br>Information Sharing*<br>Parent Participation<br>Collaboration in development	Results from pre- to post-intervention:<br><br>Improved:<br><br>Parent knowledge about CLABSI; the steps for staff to do before using a central line increased by 45 percentage points, and of time to complete "scrub the hub" by 33 percentage points.<br>Mean scores increased from "usually" to "always" for the following CLABSI prevention items:<br>a. Parents' rating of the staff's infection prevention efforts and comfort to ask staff to wash their hands.<br>b. Parent perception of dignity and respect, information sharing, participation, partnership.
Chisholm et al. (2022)[30]<br><br>U.S.	Qualitative with interviews.<br><br>100%<br><br>Pilot study.	Parents of low-income children of color in a pediatric unit of a Children's Hospital.<br><br>n=50	Inpatient navigation program led by a professional guide that included 1) education about the hospital, 2) social needs assessment, 3) cultural and communication assessment, 4) communication coaching for parents, 5) emotional support through	Qualitative Results:<br>Common themes across all domains of the framework were improved communication, feeling supported in the care environment, increased knowledge,

Authors (Year), Country	Study Design/Methods, Quality Appraisal, Analyses	Setting, Sample, Family Involvement	Intervention Description, FCC Principles (Primary Emphasis*)	Quasi-Experimental Results*, Quantitative Results, Qualitative Results
		Intervention was informed by a survey, focus groups, collaborative workshops and pilot testing that include parents.	frequent check-ins, and 6) a follow-up phone call.<br><br>Respect and Dignity*<br>Information Sharing<br>Parent Participation<br>Collaboration in development	skills and understanding, and improved knowledge of the hospital and resources.
Glick et al. (2020)[31]<br><br>U.S.	Cross-sectional survey with open-ended questions.<br><br>81%<br><br>Descriptive and comparing preference of resource by exposure to resources. Chi-square test and Fisher's exact test.	Parents of children in a pediatric unit in a Children's Hospital.<br><br>n=200	Multidisciplinary family-centered rounds (FCR) included written information and a care team that asked parents daily if they would like to attend FCR. Providers used tablets, paper notes, or a computer on wheels during FCR.<br><br>Respect and Dignity<br>Information Sharing*<br>Parent Participation	Descriptive Results:<br>Parent reported factors that affected their engagement during FCR: clear explanations from the medical team (78.5%), understanding medical information (75.5%), being dependent on health of the child (74.5%), medical team asking their input (71%).<br>Few parents believed that the type of resource used by providers during FCR (tablet, paper, or computer) affected their participation (25%).<br>56% had no preference which type of resource (tablet, paper, or computer) was used.<br>Comparative Results:<br>Among parents with a resource preference, those exposed to tablets during FCR were more likely to have a resource preference for tablets compared to those

Authors (Year), Country	Study Design/Methods, Quality Appraisal, Analyses	Setting, Sample, Family Involvement	Intervention Description, FCC Principles (Primary Emphasis*)	Quasi-Experimental Results*, Quantitative Results, Qualitative Results
				not exposed (27 percentage point increase). Exposure to computers on wheels or paper notes were not associated with preference for those resources. No parent or child characteristics were associated with preference for a specific resource.
Kelly et al. (2017)[32]<br><br>U.S.	Cross-sectional surveys with closed- and open-ended questions.<br><br>69%<br><br>Descriptive and comparing satisfaction among participants with different characteristics. Chi-square test, Somers' delta, and Mann-Whitney U test.	Parents of children in a pediatric unit.<br><br>n=90	HIT; an inpatient portal on a tablet computer that allowed parents to access to the child's records in real time including vitals, medication lists, test results, daily schedule, and education. It also allowed parents to see photos of the medical team and send them messages.<br><br>Respect and Dignity<br>Information Sharing*<br>Parent Participation	Descriptive Results:<br>296 parents used the portal, sending 176 requests and 36 messages.<br>Most used features: vital signs, medication list, healthcare team information, and schedules.<br>Parent ratings about portal ranged from 89-98% for the following: satisfied with portal, easy to use, improved access to helpful information to make decisions for their child, perceived that portal use reduced errors in care.<br>Parents found errors in their child's medication list (8%).<br>Parents reported that portal use improved health care team communication (60%).<br><br>Comparative Results:

Authors (Year), Country	Study Design/Methods, Quality Appraisal, Analyses	Setting, Sample, Family Involvement	Intervention Description, FCC Principles (Primary Emphasis*)	Quasi-Experimental Results*, Quantitative Results, Qualitative Results
				Parents with lower levels of education or who had not previously used an outpatient portal were more satisfied with the portal. Parents of children hospitalized with fever (but not other health conditions) were more positive about the portal overall and more likely to agree that the portal was useful, helped them monitor their child's health, and their child's care was better with it.
Khan et al. (2018)[38]<br><br>U.S. and Canada	Quasi-experimental comparing different groups pre and post with surveys.<br><br>82%<br><br>Multi-center study. Poisson regression estimated via generalized estimating equations. Reported unadjusted results as potential confounders were comparable between groups.	Parents of children in a pediatric unit in one of 7 hospitals.<br><br>Parents<br>Pre, n=947<br>Post, n=890<br><br>Intervention was coproduced by families, nurses, and physicians.	The I-PASS FCR program included 1) communication framework, 2) rounds report, 3) rounds training, and 4) process changes.<br><br>Respect and Dignity<br>Information Sharing*<br>Parent Participation<br>Collaboration in development	Results from pre- to post-intervention:<br>Improved:<br><br>Harmful errors decreased by 37.9%.<br>Non-preventable adverse events decreased by 58.7%.<br><br>Family centered rounds occurred 10.6% more frequently.<br>Top box ("excellent') scores increased between 8-22% for the following items: family engagement, families expressing concerns at the start of rounds, reading back plans, synthesis being completed on rounds.<br>Non-significant changes:

Authors (Year), Country	Study Design/Methods, Quality Appraisal, Analyses	Setting, Sample, Family Involvement	Intervention Description, FCC Principles (Primary Emphasis*)	Quasi-Experimental Results*, Quantitative Results, Qualitative Results
				Parent reporting of child's illness severity increased.<br>No differences:<br>Family experience scores for 19 of 25 items, including items about overall experience with hospital stay, illness severity, effective use of plain language, and frequency of teaching on rounds did not change.<br>Non-harmful errors and the overall medical error rate (the sum of harmful and nonharmful errors) did not change.
Khan et al. (2017)[33]<br><br>U.S.	Quasi-experimental comparing different groups pre and post with surveys.<br><br>73%<br><br>Multivariable linear, logistic, or repeated measures regression modeling.	Parents of children in one of two pediatric units in a children's hospital.<br><br>Pre, n=281<br>Post, n=183<br><br>Intervention was developed with input from multiple disciplines and a Family Advisory Council.	Nighttime communication intervention based on I-PASS structure that included a nurse-physician brief, a family huddle, and a family update sheet.<br><br>Respect and Dignity<br>Information Sharing*<br>Parent Participation<br>Collaboration in development	Results from pre- to post-intervention:<br>Improved:<br>Independently assessed shared understanding (having 2 of 3 congruent answers) between parent and nurse increased from 36.2% to 48.2% (adjusted).<br><br>Top-box parent experience improved for one of four domains:<br>Experience and communication with nighttime doctors increased from 23.6% to 31.5% (adjusted).<br><br>No differences:

Authors (Year), Country	Study Design/Methods, Quality Appraisal, Analyses	Setting, Sample, Family Involvement	Intervention Description, FCC Principles (Primary Emphasis*)	Quasi-Experimental Results*, Quantitative Results, Qualitative Results
				Independently assessed shared understanding between parent and resident did not change.<br><br>Top-box parent experience for the following three items did not change: Understanding of overnight plan, experience and communication with nighttime nurses, and shared understanding with and between doctors and nurses.
Krisnana et al. (2019)[42]<br><br>Indonesia	Quasi-experimental comparing different groups pre and post with surveys.<br><br>55%<br><br>Wilcoxon's signed rank test and Mann Whitney U test.	Mothers of children in a pediatric oncology floor.<br><br>Control n=30<br>Intervention n=30	The Family Centered Empowerment (FACE) Module included education sessions (2 x 30 minutes) on nutritional needs of children with leukemia, preventing infection, and preventing bleeding.<br>The control group received the standard hospital counseling through written information.<br><br>Respect and Dignity<br>Information Sharing*<br>Parent Participation	Results from pre- to post-intervention: Improved:<br><br>The intervention group mothers' mean stress scores on the DASS-21 were lower after the intervention than before (scores not reported) and lower (8.33) than the control group (12.80) after the intervention.
Leland et al. (2017)[34]<br><br>U.S.	Quasi-experimental comparing different groups pre and post; surveys on	Parents of children in a PICU of an academic quaternary care hospital.	Project ROSE (Reach Out, Soothe, and Embrace) began with multidisciplinary training for a unit-wide practice change to promote physical contact between with	Results from pre- to post-intervention: Improved:

Authors (Year), Country	Study Design/Methods, Quality Appraisal, Analyses	Setting, Sample, Family Involvement	Intervention Description, FCC Principles (Primary Emphasis*)	Quasi-Experimental Results*, Quantitative Results, Qualitative Results
	intervention days one and four.<br><br>64%<br><br>Wilcoxon rank sum test. Potential confounders were comparable between groups.	(Pre/Post)<br>Day 1: n=174/157<br>Day 4: n=46/48	parent-child. Parents received written information. During daily FCR parents and the team determined whether children were "safe to hold" (transferred to parent's arms), including those being mechanically ventilated.<br><br>Respect and Dignity<br>Information Sharing<br>Parent Participation*	Median caregiver spiritual well-being scores on FACIT-Sp-12 on day 4 increased by a median of 2, possible score 60, (effect size 0.47).<br>Percentage of caregivers who reported "quite a bit" (4) or "very much" (5) increased between 20 and 29 percentage points for the following items: Able to touch child as often as would like; Feel supported by medical team; Feel valued as a team member. Feel that child's illness does not limit physical contact.<br>No differences:<br>Median caregiver spiritual well-being scores on day one.<br>Unplanned extubation incidents.
Nankali et al. (2023)[43]<br><br>Iran	Quasi-experimental comparing mothers' scores before and 5 days after intervention, with different groups pre and post. Mothers were observed and scored.<br><br>73%	Parents of children with acute gastroenteritis in a pediatric unit.<br><br>Pre, n=80<br>Post, n=80	Written information about gastroenteritis and practical education about handwashing, giving ORS to their child, and skin care in one to three sessions for 20–35min on two consecutive days.<br><br>Respect and Dignity<br>Information Sharing*<br>Parent Participation	Results from pre- to post-intervention:<br>Improved:<br>Healthcare team observation of mothers' care practices on the Care Management of Gastroenteritis (CMG) checklist (assessing hygiene principles, fluid therapy, diet, fever, and skin care) increased from 24.47 to 78.91 (maximum score 112.) Scores were higher both within and between groups.

Authors (Year), Country	Study Design/Methods, Quality Appraisal, Analyses	Setting, Sample, Family Involvement	Intervention Description, FCC Principles (Primary Emphasis*)	Quasi-Experimental Results*, Quantitative Results, Qualitative Results
	ANOVA. Potential confounders were comparable between groups.			No differences:<br>Control group pre-test (25.98) vs. day 5 post-test (25.63).
O'Connell et al. (2017)[35]<br><br>U.S.	Mixed Methods<br>Quasi-experimental with surveys.<br>Qualitative with telephone interviews and focus groups.<br><br>60%<br><br>Descriptive only.	Parents whose children received resuscitation in one of three Level 1 trauma pediatric emergency departments.<br><br>Present, telephone survey and interview, n=99.<br><br>Not present for any reason, telephone survey and interview, n=27.<br><br>Includes subgroups:<br><br>Present, attended focus group, n=17.<br><br>Not present, attended focus group, n=8.	Three trauma centers had an FCC program which allowed parent presence during their child's resuscitation. Each family had an assigned social worker whether they were present or not. This study compared parents who were present with parents who were not (for any reason).<br><br>Respect and Dignity<br>Information Sharing<br>Parent Participation*	Descriptive Results:<br>Parent attitudes about being present 3.65 vs. wanting to be present 3.2 (4-point scale, with higher more positive.)<br>Parents who were present: self-reported positive interactions with their child, 4 items all >90%, and positive interactions with staff, 2 items >81%.<br>Parents who were present: Had trouble controlling their emotions, 8% and had to step out of the trauma room for personal reasons, 11%.<br>Parents not present: wanted to be present, 82%<br><br>Qualitative Themes:<br>The choice to be there is my right; There are limitations to being there; I can better advocate on behalf of my child; It helps to comfort my child; It gives me comfort and peace of mind; Family presence lets me see my child's care; Desire for real-

Authors (Year), Country	Study Design/Methods, Quality Appraisal, Analyses	Setting, Sample, Family Involvement	Intervention Description, FCC Principles (Primary Emphasis*)	Quasi-Experimental Results*, Quantitative Results, Qualitative Results
				time observation; Trust the staff to give the best care possible.
Pereira et al. (2021)[39]<br><br>Canada	Quasi-experimental comparing different groups pre and post with surveys.<br><br>36%<br><br>Pilot study. Descriptive only. COVID-19 pandemic interfered with post data collection, so pre-post comparison was not made.	Parents of children hospitalized in a PICU.<br><br>Volunteer program Pre, n=25<br>Post, n=5, most gave partial responses.<br>Peer mentor program Pre, n=21.<br>Post, n=6, most gave partial responses.<br><br>Intervention developed with a multi-disciplinary team including parent advisors.	A two-pronged FCC Peer and Volunteer Program to help a) families adjust to the PICU experience with the support of a peer mentor, and b) patients receive non-medical interaction from trained volunteers who had personally been hospitalized.<br>Respect and Dignity*<br>Information Sharing<br>Parent Participation<br>Collaboration in development	Descriptive results only:<br>Pre-Intervention:<br>Parents reported that volunteers would be helpful (84%), feeling comfortable with a volunteer (72%), volunteer spending time with my child (64%), and volunteer sharing ideas (60%).<br><br>Parents reported that peer mentors: would provide advice for my child's care (62%), have an understanding of my experience (62%), and would support me through this experience (52%).<br><br>Post-Intervention:<br>Volunteers had 86 interactions with 30 patients.<br>5 parents gave partial responses to surveys. Parents rated the volunteers positively and neutrally (data not reported) and one parent (20%) rated the volunteers negatively.<br>Peer mentors visited the five families 1-3 times in the past 7 days.

Authors (Year), Country	Study Design/Methods, Quality Appraisal, Analyses	Setting, Sample, Family Involvement	Intervention Description, FCC Principles (Primary Emphasis*)	Quasi-Experimental Results*, Quantitative Results, Qualitative Results
				Parents reported that a peer mentor provided support (n=4, 67%) and provided tips and advice (n=3, 50%).
Pollock et al. (2022)[36]<br><br>U.S.	Qualitative<br>Retrospective data from EHR<br><br>38%<br><br>Pilot study. Data was based on perspectives/chart notes of the family partners.	Parents of CYSHCN in inpatient units in a large healthcare system.<br>28 parents<br><br>Parents with current or past lived experience caring for CYHCN were trained to be family partners.	A parent peer support program for parents of CYSHCN that included social, emotional, cultural, and educational support.<br><br>Respect and Dignity*<br>Information Sharing<br>Parent Participation<br>Collaboration in development	Qualitative Results:<br><br>100 total supportive contacts were made in the inpatient setting.<br><br>75% of supportive contacts across settings involved emotional support, and 38% involved tangible support.<br><br>Content area of tangible support was: Educational (5%), vocational (2%), financial (12%), social recreational (0%), future planning (0%), and other (81%,) which included information about self-care, building advocacy skills related to speaking with the healthcare team, and identifying community resources.<br><br>Themes from family partners' chart notes: complexity of caring for CYSHCN (25%), stress management (38%), financial concerns (7%), health concerns

Authors (Year), Country	Study Design/Methods, Quality Appraisal, Analyses	Setting, Sample, Family Involvement	Intervention Description, FCC Principles (Primary Emphasis*)	Quasi-Experimental Results*, Quantitative Results, Qualitative Results
				(13%), and the need for parent self-care (22%).
Runaas et al. (2017)[37]<br><br>U.S.	Qualitative with interview.<br><br>50%	Parents of children hospital in a pediatric unit for BMT.<br><br>n=10<br><br>Intervention development included getting feedback from patients and caregivers in the design of the HIT prototypes.	HIT, The BMT Roadmap is a tool delivered on a tablet computer Including lab results, healthcare team directory, phases of care, discharge checklist.<br><br>Respect and Dignity<br>Information Sharing*<br>Parent Participation<br>Collaboration in development	Qualitative Results:<br><br>Caregivers found the tool useful and easy to use, leading them to want even greater access to information. BMT Roadmap was feasible, with no disruption to inpatient care.<br>Five themes:<br>1. Emotional impact of the BMT process itself.<br>2. Critical importance of communication among patients, caregivers, and healthcare providers.<br>3. Ways in which BMT Roadmap was helpful during inpatient setting.<br>4. Suggestions for improving BMT Roadmap.<br>5. Other strategies for organization and management of complex healthcare needs that could be incorporated into BMT Roadmap.
Seliner et al. (2017)[44]<br><br>Switzerland	Mixed methods<br>Quasi-experimental with historical control group using surveys.	Parents of children with profound disabilities undergoing hip surgery	Preadmission program led by an APRN that Included handouts, a counseling phone call before hospitalization, support for	Non-Significant Results from pre- to post-intervention:<br>Improved: Parent satisfaction increased by 0.46 on a 4-point scale on the MOC-20

**Authors (Year), Country**	**Study Design/Methods, Quality Appraisal, Analyses**	**Setting, Sample, Family Involvement**	**Intervention Description, FCC Principles (Primary Emphasis*)**	**Quasi-Experimental Results*, Quantitative Results, Qualitative Results**
	Qualitative with interviews.<br><br>40%<br><br>Pilot study. Fisher's exact test, *t*-test, and Mann–Whitney *U*-test. Results not adjusted for confounders despite differences between groups.	on two pediatric surgery units of a hospital.<br><br>Intervention n=14<br>Historical Control group n=14	healthcare team, and at least one visit in the hospital.<br><br>Respect and Dignity*<br>Information Sharing<br>Parent Participation	domain of general information, effect size: Cohen's d=0.35/small (p=0.388)<br>Worsened: Four other domains of MPOC-20 decreased by a change of 0.24 to 0.46 on a 4-point scale: respectful and supportive care, coordinated and comprehensive care, enabling and partnership, providing specific information (p> 0.42 or more).<br>Qualitative Results:<br><br>Continuous support: Mothers felt well prepared, informed, and able to manage the admission appropriately.<br><br>Care coordination: Parents experienced a smooth admission, but many reported that the remainder of the child's hospitalization fell short of their expectations.<br><br>Being equal partners: During the hospitalization, some mothers reported feeling disregarded, not being taken seriously, or not being appreciated for providing care for their child.

Authors (Year), Country	Study Design/Methods, Quality Appraisal, Analyses	Setting, Sample, Family Involvement	Intervention Description, FCC Principles (Primary Emphasis*)	Quasi-Experimental Results*, Quantitative Results, Qualitative Results
Uhm & Choi (2019)[40]<br><br>Korea	Longitudinal study using prospective data from the same intervention as Uhm & Kim (2019).<br><br>63%<br><br>Secondary data analysis. Repeated measures ANOVA.	Mothers of children hospitalized on a cardiac PICU.<br><br>n=36<br><br>Intervention development included in-depth interviews and pilot testing with mothers.	Mother-nurse partnership program included 30-minute education sessions twice a day, a factsheet, and designated participation activities.<br><br>Respect and Dignity<br>Information Sharing*<br>Parent Participation*<br>Collaboration in development	Quantitative Results:<br><br>Improved:<br>The mean duration of parents' care participation increased steadily from phase 1 (immediate postoperative) to phase 2 (early ventilator weaning) to phase 3 (late ventilator weaning), to phase 4 (post-extubating), to phase 5 (transfer preparation) where mothers participated in care for approximately 16, 21, 25, and 30 min, respectively.<br><br>The observed physical engagement scores (max score= 10) were 2.79, 4.36, 6.07, and 7.46 points respectively.<br><br>The observed psychological connectedness scores (max score=10) were 4.29, 5.64, 7.14, and 8.39 points, respectively.<br>Mothers' reported care-specific needs for each phase of recovery.
Uhm & Kim (2019)[41]<br><br>Korea	Quasi-experimental with control group using surveys. This was the same	Mothers and their children hospitalized on a cardiac PICU.	Mother-nurse partnership program included 30-minute education sessions twice a day, a factsheet, and designated participation activities. Control group	Results from pre- to post-intervention:<br>Improved:<br>Parental satisfaction increased between 0.7 and 1.2 points on a 6-point scale for

Authors (Year), Country	Study Design/Methods, Quality Appraisal, Analyses	Setting, Sample, Family Involvement	Intervention Description, FCC Principles (Primary Emphasis*)	Quasi-Experimental Results*, Quantitative Results, Qualitative Results
	intervention as the study by Uhm & Choi (2019).<br><br>82%<br><br>ANCOVA.	Intervention, n=36 mothers; n=36 children<br><br>Control, n=37 mothers; n=37 children<br>Intervention development included in-depth interviews and pilot testing with mothers.	received regular nursing care and a brochure on postoperative information.<br><br>Respect and Dignity<br>Information Sharing*<br>Parent Participation*<br>Collaboration in development	each domain: information, care and cure, professional attitude, parent participation, and organization. Scores ranged from 4.25 pre- to 5.45 post.<br><br>Parental self-efficacy improved on the Karitane Parenting Confidence Scale from 47.45 to 52.29 total score (max possible 60) (>40 indicates non-clinical range, <31 indicates the severe clinical range).<br><br>Perceived partnership increased between 0.68 and 1.11 points on a 5-point scale for each domain: reciprocity, professional knowledge, sensitivity, collaboration, communication, shared information, and cautiousness.<br>Scores ranged from 3.47 pre to 4.89 post.<br>Anxiety scores decreased from 45.19 to 34.31 (max score 80).<br><br>No differences:<br>Parental self-efficacy domains of support and development did not change.

Authors (Year), Country	Study Design/Methods, Quality Appraisal, Analyses	Setting, Sample, Family Involvement	Intervention Description, FCC Principles (Primary Emphasis*)	Quasi-Experimental Results*, Quantitative Results, Qualitative Results
				Time taken by infants to reach full oral feeding (with a feeding goal of 100 ml/kg/day) did not change.<br><br>Post-operative length of hospital stay did not differ.
